# Effect of Carrot Pulp Incorporation and Partial Sodium Chloride Replacement on Hybrid Burger Characteristics

**DOI:** 10.3390/foods14081400

**Published:** 2025-04-17

**Authors:** Samer Mudalal, Ibrahim Hamarsheh, Nawaf Abu-Khalaf, Fuad Al-Rimawi, Ahmed Zaazaa, Dario Mercatante, Maria Teresa Rodriguez-Estrada

**Affiliations:** 1 Nutrition and Food Technology Program, Department of Agricultural Engineering, Faculty of Veterinary Medicine and Agricultural Engineering, An-Najah National University, Nablus P.O. Box 707, Palestine; ibraheem.hamarshih@yahoo.com; 2 Department of Agricultural Biotechnology, Faculty of Agricultural Sciences and Technology, Palestine Technical University, Kadoorie (PTUK), Tulkarm P.O. Box 7, Palestine; n.abukhalaf@ptuk.edu.ps; 3 Chemistry Department, Faculty of Science and Technology, Al-Quds University, Jerusalem P.O. Box 20002, Palestine; falrimawi@staff.alquds.edu; 4 Department of Agricultural Engineering, Faculty of Veterinary Medicine and Agricultural Engineering, An-Najah National University, Nablus P.O. Box 707, Palestine; ahmadzaza@najah.edu; 5 Department of Agricultural and Food Sciences, Alma Mater Studiorum—University of Bologna, 40127 Bologna, Italy; dario.mercatante2@unibo.it (D.M.); maria.rodriguez@unibo.it (M.T.R.-E.)

**Keywords:** hybrid burger, beef meat, carrot pulp, potassium chloride, microbiological properties

## Abstract

Worldwide dietary sodium intake exceeds the recommended daily allowance, generating global interest in reducing sodium content in foods. This preliminary study aimed to evaluate the effects of decreasing sodium chloride (NaCl) levels on hybrid burger characteristics by partially replacing it with potassium chloride and carrot pulp. A total of 60 beef burger patties were divided into four treatments: A (control), 1.5% NaCl; B, 1.5% NaCl + 5% carrot pulp; C, 30% replacement of NaCl with potassium chloride (KCl) + 5% carrot pulp; D, 50% replacement of NaCl with KCl + 5% carrot pulp. Carrot pulp significantly influenced color indices and pH. The control (treatment A) exhibited the lowest lightness (L*) values (31.70 vs. 40.9, 38.67, and 38.44 for treatments B, C, and D, respectively; *p* < 0.05). Additionally, carrot pulp positively affected water-holding capacity, but it led to an increase in total aerobic bacterial count by approximately 2 logs and fungal count increased by about 4 logs (cfu/g). Sensory attributes were not impacted by the addition of carrot pulp; however, replacing 50% of NaCl with KCl significantly increased bitterness. In conclusion, replacing 30% of NaCl with KCl while incorporating carrot pulp was feasible without compromising sensory properties of the hybrid burger.

## 1. Introduction

Currently, there is growing global concern regarding the health risks associated with high dietary sodium intake. Excessive sodium consumption increases the risk of several chronic diseases, including hypertension, renal failure, and cardiovascular disease [1]. A recent clinical study reported that global sodium intake averages between 9 and 12 g of sodium chloride (NaCl) per day, equivalent to approximately 3.6 to 4.8 g of sodium per day [2]. It has been found that approximately 75% of sodium intake comes from processed foods [3], with meat products contributing about 20–30% of daily sodium intake [4,5]. Additionally, Matthews and Strong [6] reported that meat and meat products rank second in dietary sodium contribution, following cereal products. Reducing sodium intake to a certain level can have a positive impact on human health. In this context, cardiovascular diseases are among the costliest health-related conditions worldwide, accounting for approximately 11% of total healthcare expenditures [7]. Both hypertensive individuals and those with normal blood pressure can benefit from reduced sodium intake, but hypertensive patients can experience even greater advantages [3]. Several studies have investigated the effects of NaCl reduction on sensory, textural, and microbiological properties of meat products, as well as on technical parameters such as yield and water-holding capacity [8,9,10]. In this context, it was found that there was a synergy between salt replacement and cellulose addition, suggesting that this combination may be useful in creating healthier pork sausages without impairing the quality [8]. Various strategies have been proposed to reduce sodium intake and sodium content in meat products. It has been demonstrated that KCl is an effective salt substitute due to its chemical and physical properties, which are similar to those of NaCl to some extent. However, at high concentrations, KCl can impart a metallic or bitter taste [11]. Mudalal and Petracci [9] showed that replacing 45% of NaCl with potassium chloride (KCl) in bicarbonate-marinated turkey breast meat did not adversely affect the quality attributes, such as texture, composition, and sensory properties. Yim et al. [10] demonstrated that the quality properties (such as texture, color, and sensory profile) of low-sodium nitrite salami were affected when NaCl was substituted with KCl and magnesium chloride (MgCl_2_).

Besides the reduction of NaCl, another priority of the meat industry is to promote clean label products using agri-food by-products to encourage circularity as stated in the United Nations SDGs 2 and 12 [12]. One example of a relevant agri-food by-product is carrot pulp, which represents about 30–50% of carrot after juice production [13]. The latter is a widely consumed beverage in Palestine due to its affordability and numerous health benefits given its rich nutritional content. Carrot pulp is known to be rich in essential nutrients, including proteins, sugars, minerals (K, Ca, Mg, etc.), vitamins (Vit A and Vit C) and fats [14]; in particular, it is a valuable source of carotenoids and dietary fiber, which provide support for the health of eye and heart [13]. Thus, the incorporation of carrot pulp in meat products may enhance their nutritional profile and texture. Furthermore, carrot pulp may contribute to NaCl replacement and lean meat reduction. Therefore, the primary objective of this preliminary study was to evaluate the feasibility of reducing NaCl content in beef burgers by partially replacing it with KCl while incorporating carrot pulp.

## 2. Materials and Methods

### 2.1. Sample Preparation

Beef meat was obtained from a local meat shop in Tulkarm (Palestine). Fat, connective tissue, and cartilage were removed from the muscle. The meat was minced with a 3 mm plate in a meat grinder. The minced meat was thoroughly blended to achieve a homogeneous composition and microbial load. The total amount of minced meat was 6 kg, and it was separated into 4 portions (each portion 1.5 kg) corresponding to four treatments as described in Table 1. Carrot pulp is a by-product obtained during the carrot juice extraction process. It was collected from a local juice shop in Tulkarem City (Palestine). The collection of carrot pulp was under our researcher’s supervision. The carrots were thoroughly cleaned with water before juice extraction. The carrot pulp was used as fresh raw material for the preparation of beef patties, without subjecting it to any stabilization/sanitization pre-treatment.

Patties (100 g each) were prepared for every treatment, resulting in 15 patties per treatment and 60 patties in total. The meat patties were frozen at −18 °C until analysis. Samples were subjected to the following analysis: color (L*a*b*), pH, water-holding capacity (cooking loss and swelling), texture profile (hardness, cohesiveness, gumminess, springiness, and chewiness), microbiological analysis (total aerobic plate count, yeasts, and molds), and sensory analysis (hedonic).

### 2.2. Color Indices

Color indices (L*a*b*) were measured according to the CIE (Commission Internationale de l’Eclairage) system using a chromameter (Minolta Chroma Meter CR-400, Tokyo, Japan) and a light source. Eight samples were selected from each group to measure color characteristics. Three uniform areas (free from color defects and anomalies on the surface of the meat patties) were selected from each sample for color measurements, and the average value was recorded. To more thoroughly examine the color changes in the hybrid burgers, chroma (C*) was calculated using equation $\sqrt{\left(a\;*\right)2+\left(b\;*\right)2}$, and total color difference (ΔE) was calculated using an equation $\sqrt{\left(\Delta L\;*\right)2+\left(\Delta a\;*\right)2+\left(\Delta b\;*\right)2}$ where the control was considered as a reference.

### 2.3. pH Measurements

Eight samples from each group were chosen to measure pH. About 2.5 g of minced meat was transferred to 25 mL of distilled water and then blended with an Ultra-turrax (Staufen, Germany) to obtain a homogeneous milky suspension (60 s). For calibration of the pH meter (IQ Scientific Instruments, San Diego, CA, USA) before sample measuring, 4.0 and 7.0 pH buffer solutions were used.

### 2.4. Microbiological Analysis

Microbiological assessment was carried out for each experimental group by estimating the count of total aerobic bacteria as well as yeast and mold.

The total aerobic bacterial and fungal counts were determined in 8 replicates. The first dilution was prepared by transferring 10 g of minced meat in aseptic conditions to 90 mL peptone water solution. In order to obtain a proper count using culture media, five dilutions (10^−1^ to 10^−5^) were employed to estimate the count of bacteria, mold, and yeast.

Plate count agar (PCA) was used as growth medium for aerobic bacteria to determine total aerobic bacterial count. The plates were held in the incubator for 48–72 h at 37 °C. A 10 g of sample of beef patties was homogenized using a stomacher with 90 mL of 0.1% peptone water to create a uniform suspension. Proper serial dilutions were prepared to obtain a countable range. Potato dextrose agar (PDA), with a pH 3.5–5.0, was used to culture the fungi in the sample by spreading 0.1 mL of the diluted sample. The plates were incubated at 20–25 °C for 5–7 days. After incubation, the plates were examined for visible mold colonies. Mold colonies are typically fuzzy, powdery, or filamentous and may vary in color (e.g., white, green, black, blue). The remaining colonies were considered as yeasts. The results were recorded as the sum of both the fungi and yeast counts.

### 2.5. Sensory Analysis

The samples were evaluated by 29 untrained consumers from both genders (aged 18–25 years). Consumers were chosen based on their willingness to participate in the study and their frequent consumption of beef burgers. The consumers were informed about the purpose of the study and all relevant procedures regarding the sensory analysis. The Institutional Review Board (IRB) of An-Najah National University examined and authorized the sensory analysis with approval number (Agr. April 2024/10).

Four beef burger patties were selected per group and cooked in an electric oven (200 °C for 15 min), turning the patties four times during cooking. Thereafter, the cooked patties were allowed to cool at room temperature and divided into small, uniform pieces (8 pieces/patty). Samples were offered in plastic plates and tasted by panelists in booths, who carried out a liking test and rated the sensory attributes using a 9-point scale (9 = Like Extremely; 1 = Dislike Extremely). Various sensory attributes were evaluated (saltiness, cohesiveness, color acceptability, metallic taste, odor acceptability, bitter taste, flavor acceptability, texture acceptability, flavor acceptance, general acceptability).

### 2.6. Water-Holding Capacity (WHC)

The ability of the meat to bind or retain water was estimated by measuring water released after centrifugation. The centrifuge speed was set at 7500 rpm. After centrifugation, the supernatant layer in the test tubes was removed, and then the contents of the test tubes were weighed [15]. Five replications for each treatment were considered for this analysis.

$$WHC=\frac{weight\;of\;sample\;before\;centrifugation\left(g\right)-weight\;after\;supernatant\;removal\;(g)}{meat\;sample\;mass\;(g)}\times 100$$

### 2.7. Cooking Loss

Six beef patties representing each treatment were weighed before and after cooking (the same patties that were used for sensory analysis). Cooking loss was determined using the following formula:

$$\%\;Cooking\;loss=\frac{\left(weight\;before\;cooking-weight\;after\;coking\right)}{\left(weight\;before\;cooking\right)}\times 100$$

### 2.8. Textural Profile Analysis (TPA)

Texture profile analysis (including hardness, cohesiveness, springiness, gumminess, chewiness, and cohesiveness) was performed using a texture analyzer (Brookfield Texture Analyzer CT50, Brookfield Engineering Laboratories, Middleboro, MA, USA). Six samples from each treatment were cut into a cylindrical shape with a 20 mm diameter and 10 mm height. During the TPA measurements, 40% of the sample height was compressed using a 50 kg load cell connected to a cylindrical aluminum probe of 50 mm DIA. The velocity of the probe during the test was 2 mm/s, while the velocities before and after the test were both 2 mm/s.

### 2.9. Statistical Analysis

All results were subjected to a one-way analysis of variance (ANOVA) using SPSS software (Version 21, IBM Corporation, Armonk, NY, USA) in order to ascertain the impact of using KCl and carrot pulp for burger formulation, instead of NaCl. The chosen *p*-value for statistical significance was <0.05. The means of quality traits that differed statistically were separated using Duncan’s multiple range test.

## 3. Results and Discussion

Hybrid patties with reduced NaCl content were formulated according to the WHO guidelines on salt consumption (<5 g/day salt in adults) [2], thus contributing to 15–21% of the WHO recommended daily salt consumption. The effects of NaCl replacement plus the addition of carrot pulp on the color indices are shown in Table 2. In general, significant differences in color indices were observed among treatments. In this context, the control treatment (A) exhibited significantly lower lightness (L*) values (31.70 vs. 40.9, 38.67, and 38.44, *p* < 0.05) compared to treatments B, C, and D, respectively, while no significant differences were found between treatments C and D. Treatments A and C had significantly lower a* values than treatments B and D. No significant differences in a* values were observed between treatments A and C or between treatments B and D. Treatment A displayed a significantly lower yellowness index (b*) compared to the other treatments, whereas treatment B showed the highest b* value. No significant differences in b* values were observed between treatments C and D.

In general, the color indices of processed meat products are influenced by several factors, such as pH, heat treatment, atmospheric composition, and the ingredients used in the products, among others [16]. The color differences observed in our study can be attributed to the use of carrot pulp, which contains several pigments, the most important of which is β-carotene [13]. In fact, the addition of carrot pulp to meat batter resulted in an increase in both redness and yellowness values [17]. Similar results were also found when carrot was added to fresh turkey sausage [18].

Color saturation or intensity is measured using chroma (C*). Treatment A had the lowest C* value (9.76), which was significantly lower than the other treatments. Treatment D showed the highest C* (12.62), while treatments B and C had similar values (around 11.7). This suggests that, compared to the control (A), the addition of carrot pulp (B) increases chroma. Chroma increased even further when NaCl was partially replaced with KCl (C and D), particularly at the 50% replacement level (D). This effect may be attributed to the additional pigments from the carrot pulp and the influence of KCl on color stability. In fact, substituting NaCl by KCl may alter protein–water interactions, improving surface reflectivity and pigment retention [5,6].

ΔE quantifies the color difference with respect to the reference (control treatment A). ΔE values decreased from 9.63 to 5.88 across treatments B, C, and D, indicating that the color gradually diverges from the control with the addition of carrot pulp and the replacement of NaCl with KCl. Treatment B had the highest ΔE (9.63), and treatment D had the lowest (5.88). The carotenoids present in carrot pulp likely enhanced chroma by increasing redness (a*) and yellowness (b*) [17]. As aforementioned, the protein–water interactions may have been modified when replacing NaCl by KCl, which could have affected surface reflectivity and pigment retention [5,6].

The effects of the treatments on pH, water-holding capacity (WHC), and cooking loss are shown in Table 3. Regarding pH, all treatments displayed a significant decrease with respect to the control (treatment A, pH 5.45). This suggests that the pH of the burgers was significantly impacted by the inclusion of both carrot pulp and KCl. In fact, the lowest pH (5.18) was found in treatment D, followed by treatments C, B, and A. The high content of organic acids (especially citric and caffeic acids) in carrot pulp [19] may explain the pH decrease observed in the hybrid burgers. Several authors have reported similar effects on pH when carrot derivatives had been added to meat products [18,20,21]. All previous studies explained the effect of carrot pulp on pH due to presence of organic acids (e.g., malic acid, citric acid) and minerals which can act as buffers or acidulants.

On the other hand, the substitution of NaCl with KCl has also been associated with a pH decrease, an effect that was observed when comparing treatment B with treatments C and D. These results agree with Lyon et al. [22], who found that the addition of NaCl to meat significantly raised the pH.

Regarding WHC, treatment A showed the lowest WHC (15.34%), while treatment B exhibited the highest one (24.77%). No significant differences in WHC were detected between C and D, but both had higher WHC than the control sample. The observed WHC of the hybrid samples might have been influenced by the addition of both carrot pulp and KCl. On one hand, carrot pulp has a high content of dietary fiber (55%), which can effectively hold water and favor an increase in WHC; however, the WHC properties of carrot fibers are known to depend on the degree of physical degradation of the fiber, as well as on the ratio of insoluble/soluble fiber [23]. On the other hand, the addition of chloride salts modifies meat pH, which affects the WHC of proteins through mechanisms involving electrostatic interactions and structural modifications. In general, higher pH values generally enhance WHC due to increased negative charges and swelling of protein structures, while lower pH values can lead to reduced water retention due to increased positive charges and tighter packing of proteins at their isoelectric points [24]. In the current investigation, the pH drops caused by the inclusion of carrot pulp in the hybrid patties was not able to impair the increase in WHC promoted by the presence of carrot fiber and chloride salts.

Concerning cooking loss, there was a gradual increase from treatment A (32.34%) to treatment D (42.46%). However, there were no significant differences in cooking loss between treatments C and D. The increase in cooking loss due to the addition of carrot pulp may be attributed due to different factors. In this context, carrot pulp has a high moisture content, so water can be easily released due to degradation of pectin, cellulose, and hemicellulose during cooking. Moreover, carrot powder has the potential to alter the protein matrix and to lower pH when added to meat products, thus increasing cooking loss [20,25]. On other hand, replacing NaCl with KCl resulted in a significant increase in cooking loss in treatments C and D compared to treatment B (without replacement). This result agrees with previous results [5,11].

The effects of the different treatments on the texture profile (hardness, springiness, gumminess, chewiness, and cohesiveness) are shown in Table 4. Our findings showed that the control sample exhibited the highest hardness value (4813.33 vs. 2781.67, 1770.00, and 1885 kg, *p* < 0.05) compared to treatments B, C, and D, respectively. Treatment A had a significantly higher springiness value than treatment B, but no significant differences in springiness were found between treatments C and D. Regarding gumminess, treatment A exhibited the highest value compared to treatments B, C, and D, while no significant differences were observed among treatments B, C, and D. No significant differences in chewiness or cohesiveness values were observed among the treatments. Several previous studies have shown that the reduction of NaCl significantly affects the texture profile of meat. In this regard, Aliño et al. [26] found that replacing 70% of NaCl with KCl in dry-cured bacon led to a reduced hardness. Li et al. [27] attributed the reduction in hardness in low-sodium cured meat to an intense protease activity, leading to a high proteolysis level. Horita et al. [28] showed that substituting 50% of NaCl with KCl in Frankfurt sausages resulted in higher hardness values compared to the control group. On the other hand, it has also been reported that the addition of carrot derivatives to meat products can also affect their texture. In fact, Öztürk-Kerimoğlu [17] found that the inclusion of carrot powder in meat batter reduced hardness, gumminess, and chewiness; these results were partially in agreement with our findings, which might depend on the physical form (i.e., slices, powder) and water content of the carrot ingredient. Other studies have shown that the addition of carrot increased the texture and hardness of products. This is attributed to the fiber content, which enhances the binding capacity of the product [29,30]. Overall, the effect of carrot derivatives on the final texture of meat products is greatly influenced by the level of milling, moisture content, and interaction of carrot components with other meat components [8,21,30].

The interaction between chloride salts and carrot fiber in meat products involves complex physicochemical mechanisms that influence protein behavior, gelation, and water dynamics. Chloride ions (Cl⁻) from salts, such as NaCl or KCl, increase the net negative charge on myofibrillar proteins, shifting the isoelectric point from ~5.2 to ~4.8 [31]. This shift enhances protein solubility and swelling, facilitating the formation of a cohesive gel network by myosin heads, which effectively traps water and fat. Monovalent salts (Na⁺, K⁺) exhibit superior myosin solubilization compared to divalent salts (Ca^2^⁺, Mg^2^⁺), resulting in stronger gels with higher cooking yields. Specifically, NaCl increases the α-helix content in myosin, stabilizing its secondary structure and promoting the formation of an ordered gel matrix. In contrast, KCl and divalent salts reduce α-helix content, leading to weaker gels. This structural stabilization is crucial for pressure-induced gelation, where a minimum of 1.2% NaCl is required to achieve optimal texture and water retention in chicken gels [32]. Carrot fiber, rich in insoluble dietary fibers such as cellulose, competes with proteins for water binding. While fibers enhance moisture retention, excessive levels (>10%) can disrupt the protein matrix, reducing cooking yield and emulsion stability. However, at optimal concentrations (approximately 10%), fibers can act as a scaffold, reinforcing the gel structure without compromising the texture [33].

The introduction of fibers into the protein network creates structural discontinuities, potentially reducing gel hardness and cohesiveness. Nevertheless, studies indicate that moderate fiber levels induce minimal texture changes, suggesting a balance between fiber water-binding capacity and protein cross-linking. The addition of chloride salts increases ionic strength, which may enhance fiber hydration and swelling. While this can improve water retention, excessive ionic strength risks inducing phase separation [33]. Moreover, salt addition alters meat batter pH (e.g., KCl > NaCl), which in turn influences fiber–protein electrostatic interactions [32]. Higher pH levels promote protein–fiber repulsion, potentially weakening the gel unless counterbalanced by ionic cross-linking mechanisms [32]. While these mechanisms align with individual studies, direct investigations on salt-fiber interactions remain limited. Critical gaps persist in understanding how ionic strength and fiber type jointly modulate protein denaturation, gel microstructure, and sensory attributes.

**Figure 1:**
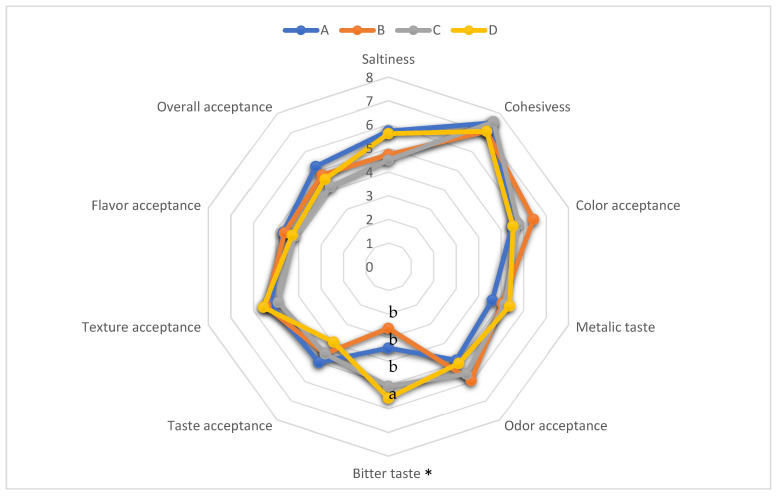
Sensory profiles of low-sodium hybrid burgers formulated with carrot pulp and KCl. Treatment A (control), 1.5% NaCl; treatment B, 1.5% NaCl and 5% carrot pulp; treatment C, 30% of NaCl was replaced with potassium chloride (1.05% NaCl, 0.45% KCl, and 5% carrot pulp); treatment D, 50% of NaCl was substituted with KCl (0.75% NaCl, 0.75% KCl, and 5% carrot pulp). * Significant differences at *p* < 0.05. ^a,b^ Means with different superscript letters were statistically different (*p* < 0.05).

Figure 1 reports the effects of the various treatments on the patties’ sensory traits. In general, there were no significant differences among treatments, except for the bitter taste. Treatment D had the highest bitter taste value compared to the other treatments, while no significant differences in this trait were found among A, B, and C. In general, the effect of reducing NaCl on sensory traits depends on the replacement level and the type of food. It was observed that the substitution of NaCl with KCl in dry-cured bacon led to an increase in juiciness and bitterness [27,34]. Gelabert et al. [35] reported that replacing 50% of NaCl with KCl had no effect on the flavor of fermented sausages. Other researchers found that 50% NaCl substitution with KCl significantly resulted in a reduction of saltiness in rabbit loin meat [36]. However, the bitterness in meat products can be masked using flavor enhancers, such as lactate salts [11], amino acids [37], yeast extracts [38], or mushroom-derived extracts [39].

On the other hand, the effects of carrot inclusion on the sensory characteristics of meat products depend on the type of raw meat as well as the chemical composition and formulation of the finished products [40,41]. For instance, Bhosale et al. [42] found no significant differences in the sensory characteristics of carrot-enriched chicken nuggets as compared to the control; however, the flavor score decreased when the carrot inclusion was higher than 10%. Eim et al. [43] reported that the addition of 3% carrot fiber to dry fermented sausage decreased the product acceptability. On the other hand, Kaur et al. [30] formulated chicken nuggets by adding carrot pulp and demonstrated that the incorporation of 10% carrot pulp did not alter the products’ sensory characteristics, while improving fiber content, reducing fat content, increasing mineral content, and lowering production costs. Regarding color, Chomanov et al. [44] found that canned goat meat with added carrot exhibited higher scores than the control; similar results have been observed in several previous studies on chicken nuggets [42] and turkey meat sausages [45]. Concerning juiciness and tenderness, the addition of carrots enhanced these texture traits in fresh beef sausages, which was attributed to the high content of fiber and polysaccharides of carrots, as these food components have a strong ability to bind and hold water in the meat product [46,47]. It was found that inclusion of carrot pomace in beef patties had a great impact on the taste and texture [23].

**Figure 2:**
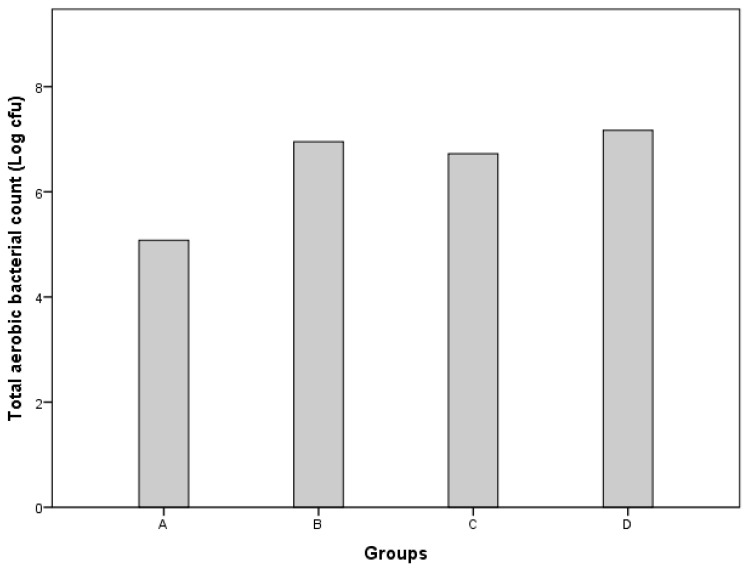
Total bacterial counts of beef patties from different treatments. Treatment A (control), 1.5% NaCl; treatment B, 1.5% NaCl and 5% carrot pulp; treatment C, 30% of NaCl was replaced with KCl (1.05% NaCl, 0.45% KCl, and 5% carrot pulp); treatment D, 50% of NaCl was substituted with KCl (0.75% NaCl, 0.75% KCl, and 5% carrot pulp).

Figure 2 shows the effects of the different treatments on the total aerobic count of the patties. The total aerobic count in carrot pulp was about 2 log cfu/g. The control sample had a significantly lower total aerobic count (5.08 vs. 6.95, 6.72, and 7.17 log (cfu/g), *p* < 0.05) than treatments B, C, and D, respectively. However, no significant differences in total aerobic count were observed among treatments B, C, and D. The increase in aerobic bacterial count in treatments B, C, and D was about 2 logs (cfu/g), which might be attributed to the addition of carrot pulp, as it was almost equivalent to the initial bacterial count detected in raw carrot pulp. On the other hand, no significant differences in total aerobic count were observed among treatments B, C, and D. These results evidence that the NaCl replacement with KCl did not significantly affect the preservative action toward total aerobic microbiota. Some researchers have reported that replacements up to 45% had no significant impact on water activity [36], one of the main parameters affecting microbial growth.

**Figure 3:**
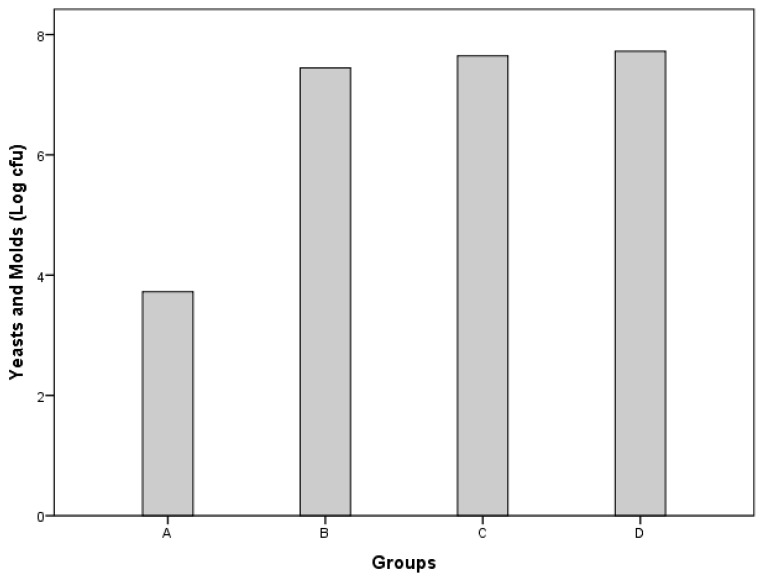
Total fungal count (yeasts and molds) of beef patties from different treatments. Treatment A (control), 1.5% NaCl; treatment B, 1.5% NaCl and 5% carrot pulp; treatment C, 30% of NaCl was replaced with KCl (1.05% NaCl, 0.45% KCl, and 5% carrot pulp); treatment D, 50% of NaCl was substituted with KCl (0.75% NaCl, 0.75% KCl, and 5% carrot pulp).

Figure 3 exhibits the effects of the various treatments on the yeast and mold counts of the hamburgers. The trends were similar to those recorded for the total aerobic count. In fact, treatment A had a significantly lower yeast and mold count (3.72 vs. 7.44, 7.65, and 7.72 log (cfu/g), *p* < 0.05) than treatments B, C, and D, respectively. No significant differences in total fungal count were noted among treatments B, C, and D. As previously stated for the total aerobic count, the increase in the count of yeasts and molds in treatments B, C, and D may be attributed to the addition of fresh carrot pulp, whose total fungal count was about 4 logs (cfu/g). In fact, the difference in fungal count between burgers with and without carrot pulp was about 4 logs (cfu/g). The absence of significant differences in fungal counts among the different treatments containing carrot pulp (B, C, and D) further confirms that the increase in fungal count is correlated to the addition of carrot pulp.

Our data disclose a relevant impact of the use of fresh carrot pulp on the microbial quality of hybrid patties, evidencing the need to carry out a sanitization pre-treatment of the fresh material with mild heat treatments (blanching, drying) or non-thermal treatments (such as high-pressure processing (HPP) or UV), which would help reduce the microbial load and improve the shelf life, while preserving the nutritional and functional properties of the pulp [48]. Moreover, the hurdle processing concept could be also useful in this context by employing natural antimicrobial agents (such as organic acids, plant extracts, essential oils, or lactate salts) while keeping a clean-label approach [1]. Another option could be to perform a sanitization treatment on the packed, finished product (i.e., HPP) [48].

## 4. Conclusions

The present study intended to formulate healthier, clean-label, and sustainable meat products that were in alignment with the international initiatives to lower sodium dietary intake to reduce the risk of hypertension and cardiovascular diseases. The results of our research, in fact, demonstrate the feasibility of replacing NaCl content with KCl (up to 30%) in hybrid beef burgers formulated with carrot pulp, without significantly compromising their sensory properties. The incorporation of carrot pulp positively influenced the water-holding capacity and color of the patties, but it negatively affected the microbiological properties, since there was an increase in both total aerobic bacterial and fungal counts, likely due to the inherent microbial load of fresh carrot pulp.

These findings suggest that a moderate reduction in NaCl, combined with the inclusion of carrot pulp, can be a viable strategy to improve the nutritional profile of meat products while maintaining consumer satisfaction. However, further studies are necessary to investigate the possible processing interventions (such as UV treatment or high hydrostatic pressure) to mitigate the microbial load associated with fresh carrot pulp and thus ensure food product safety.

## Figures and Tables

**Table 1:** Composition of beef burger formulations in all groups.

	Group A	Group B	Group C	Group D
Composition (g/100 g)				
Minced meat	98.5	93.5	93.5	93.5
Carrot pulp	-	5	5	5
NaCl	1.5	1.5	1.05	0.75
KCl	-	-	0.45	0.75

**Table 2:** Effect of NaCl substitution by KCl and addition of carrot pulp on hamburger color indices (L*a*b*).

	Treatments				
	A	B	C	D	*p*-Value
L*	31.70 ± 2.74 ^c^	40.9 ± 1.39 ^a^	38.67 ± 1.56 ^b^	38.44 ± 2.18 ^b^	<0.05
a*	7.70 ± 0.54 ^b^	8.43 ± 0.241 ^a^	7.43 ± 0.73 ^b^	8.50 ± 0.40 ^a^	<0.05
b*	6.05 ± 0.67 ^c^	9.56 ± 0.36 ^a^	8.67 ± 0.41 ^b^	8.96 ± 0.54 ^b^	<0.05
C*	9.76 ± 0.37 ^c^	11.74 ± 0.40 ^b^	11.64 ± 0.45 ^b^	12.62 ± 1.12 ^a^	<0.05
ΔE		9.63 ± 0.51 ^a^	6.87 ± 1.21 ^b^	5.88 ± 2.7 ^b^	<0.05

Data were reported as means ± standard deviation. ^a–c^ Means in the same row with different superscript letters were statistically different (*p* < 0.05). Treatment A (control), 1.5% NaCl; treatment B, 1.5% NaCl and 5% carrot pulp; treatment C, 30% of NaCl was replaced with KCl (1.05% NaCl, 0.45% KCl, and 5% carrot pulp); treatment D, 50% of NaCl was substituted with KCl (0.75% NaCl, 0.75% KCl, and 5% carrot pulp).

**Table 3:** Effect of NaCl substitution with KCl and the addition of carrot pulp on hamburger pH, swelling, and cooking loss.

	Treatments				
	A	B	C	D	*p*-Value
pH	5.45 ± 0.01 ^a^	5.28 ± 0.02 ^b^	5.24 ± 0.04 ^c^	5.18 ± 0.01 ^d^	<0.05
WHC	15.34 ± 5.88 ^b^	24.77 ± 3.26 ^a^	19.93 ± 1.76 ^c^	21.26 ± 1.54 ^c^	<0.05
Cooking loss	32.34 ± 1.38 ^c^	36.34 ± 3.11 ^bc^	40.17 ± 0.35 ^ab^	42.46 ± 2.88 ^a^	<0.05

Data were reported as means ± standard deviation. ^a–d^ Means in the same row with different superscript letters were statistically different (*p* < 0.05). Treatment A (control), 1.5% NaCl; treatment B, 1.5% NaCl and 5% carrot pulp; treatment C, 30% of NaCl was replaced with KCl (1.05% NaCl, 0.45% KCl, and 5% carrot pulp); treatment D, 50% of NaCl was substituted with KCl (0.75% NaCl, 0.75% KCl, and 5% carrot pulp).

**Table 4:** Effect of NaCl replacement with KCl and the addition of carrot pulp on hamburger texture profile (hardness, springiness, gumminess, chewiness, and cohesiveness).

	Treatments				
	A	B	C	D	*p*-Value
Hardness	4813.33 ± 543.13 ^a^	2781.67 ± 627.54 ^b^	1770.00 ± 114.601 ^b^	1885.00 ± 747.63 ^b^	<0.05
Springiness	9.66 ± 1.85 ^a^	5.76 ± 1.95 ^b^	0.78 ± 0.07 ^c^	0.75 ± 0.05 ^c^	<0.05
Gumminess	3421.40 ± 404.2 ^a^	1838.00 ± 494.75 ^b^	1378.75 ± 563.10 ^b^	1402.00 ± 755.84 ^b^	<0.05
Chewiness	327.70 ± 145.21	187.80 ± 70.54	188.70 ± 386.79	189.18 ± 125.13	0.10
Cohesiveness	0.66 ± 0.10	0.64 ± 0.12	0.67 ± 0.09	0.57 ± 0.25	0.67

Data were reported as means ± standard deviation. ^a–c^ Means in the same row with different superscript letters were statistically different (*p* < 0.05). Treatment A (control), 1.5% NaCl; treatment B, 1.5% NaCl and 5% carrot pulp; treatment C, 30% of NaCl was replaced by KCl (1.05% NaCl, 0.45% KCl, and 5% carrot pulp); treatment D, 50% of NaCl was substituted by KCl (0.75% NaCl, 0.75% KCl, and 5% carrot pulp).

## Data Availability

The data presented in this study are available on request from the corresponding author.

## Abbreviations

The following abbreviations are used in this manuscript:

WHC	Water-Holding Capacity
PCA	Plate Count Agar
PDA	Potato Dextrose Agar
CFU	Colony Forming Unit
TPA	Textural Profile Analysis
ANOVA	Analysis of Variance
